# Modelling that shaped the early COVID-19 pandemic response in the UK

**DOI:** 10.1098/rstb.2021.0001

**Published:** 2021-07-19

**Authors:** Ellen Brooks-Pollock, Leon Danon, Thibaut Jombart, Lorenzo Pellis

**Affiliations:** ^1^ Bristol Veterinary School, University of Bristol, Bristol BS40 5DU, UK; ^2^ NIHR Health Protection Research Unit (HPRU) in Behavioural Science and Evaluation, Population Health Sciences, Bristol Medical School, University of Bristol, Bristol BS8 2BN, UK; ^3^ Department of Engineering Mathematics, University of Bristol, Bristol BS8 1TW, UK; ^4^ Centre for Mathematical Modelling of Infectious Diseases, Department of Infectious Disease Epidemiology, London School of Hygiene and Tropical Medicine, London WC1E 7HT, UK; ^5^ MRC Centre for Global Infectious Disease Analysis, Department of Infectious Disease Epidemiology, School of Public Health, Imperial College London, UK; ^6^ Department of Mathematics, University of Manchester, Manchester M13 9PL, UK; ^7^ The Alan Turing Institute, London, UK

**Keywords:** infectious disease modelling, modelling for policy, COVID-19

## Abstract

Infectious disease modelling has played an integral part of the scientific evidence used to guide the response to the COVID-19 pandemic. In the UK, modelling evidence used for policy is reported to the Scientific Advisory Group for Emergencies (SAGE) modelling subgroup, SPI-M-O (Scientific Pandemic Influenza Group on Modelling-Operational). This Special Issue contains 20 articles detailing evidence that underpinned advice to the UK government during the SARS-CoV-2 pandemic in the UK between January 2020 and July 2020. Here, we introduce the UK scientific advisory system and how it operates in practice, and discuss how infectious disease modelling can be useful in policy making. We examine the drawbacks of current publishing practices and academic credit and highlight the importance of transparency and reproducibility during an epidemic emergency.

This article is part of the theme issue ‘Modelling that shaped the early COVID-19 pandemic response in the UK’.

## 1. Introduction

The year 2020 will be remembered as the year of the COVID-19 pandemic. On 31 December 2019, a cluster of cases of pneumonia was reported in Wuhan, China. A few weeks later human-to-human transmission was confirmed. By the end of January 2020, the World Health Organization reported 7818 confirmed cases in 18 countries, with the majority in China [1]. The infection spread rapidly around the world, with a large early outbreak in Italy. By the end of the year, there had been 85 million confirmed cases, 1.8 million deaths and unprecedented movement bans and social distancing.

On the last day of January 2020, two COVID-19 cases were confirmed in the UK [2]. The number of cases grew slowly but steadily. On 16 March 2020, the Prime Minister Boris Johnson announced that ‘according to SAGE, it looks as though we're now approaching the fast growth part of the upward curve. And without drastic action, cases could double every 5 or 6 days' [3, lines 10–13]. From 23 March 2020, all non-essential contact with others and unnecessary travel were prohibited, and this order stayed in place until schools were partially re-opened on 1 June 2020.

This theme issue contains some of the modelling behind policy decisions in the UK. The authors are contributors to the Scientific Pandemic Influenza Group on Modelling-Operational (SPI-M-O), the Scientific Advisory Group for Emergencies (SAGE) subgroup that provides modelling expertise. Here, we discuss the UK scientific advisory system and how it operates in practice, how and why infectious disease modelling is useful in policy making, drawbacks of current publishing practices and the papers contained in this special issue.

## 2. The UK science advisory system

Science is an integral part of the evidence that is considered when developing government policy. The UK government and civil service has a structure for receiving both routine and emergency scientific advice. The two scientists at the centre of the UK government are the Government Chief Scientific Advisor (GCSA), currently Patrick Vallance, and the Chief Medical Officer (CMO), currently Chris Whitty. Most government departments also have a Chief Scientific Advisor with specific knowledge of the area. Scientific Advisors are typically well-established university academics.

As well as Scientific Advisors, the GCSA and CMO chair SAGE. SAGE draws on expertise from multiple fields relevant to the given emergency. SAGE has been activated nine times since 2009, for example in 2019 in response to the potential breach of the Toddbrook reservoir [4]. On 22 January 2020, SAGE met for the first time to discuss the emerging novel coronavirus [5]. SPI-M-O, composed principally of infectious disease modellers [6], was convened in 2009 for H1N1 influenza and in 2014 in relation to Ebola. It first met on 27 January 2020 to discuss COVID-19. SPI-M-O met at least weekly for the duration of 2020 and has continued in 2021. Its membership has expanded to around 50 modellers from multiple universities and Public Health England [7].

Much of the early SPI-M-O work involved estimating key epidemiological parameters and drivers, such as the growth rate, the incubation period and the mortality rate. SPI-M-O produces weekly consensus estimates of the growth rate and the reproduction number [8] as well as short- and medium-term projections. Lastly, SPI-M-O responds to policy-specific questions, for example, exploring the likely impact of support bubbles or contact tracing and producing scenarios prior to policy changes, like reopening schools or entering and exiting from lockdown [5].

**Figure 1.**
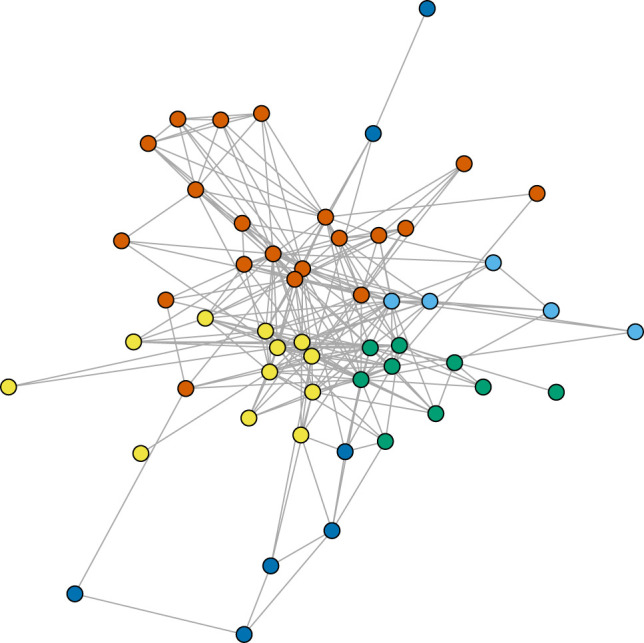
Collaboration network for SPI-M-O contributors. Graph created from PubMed results on 23 March 2021 with the list of SPI-M contributors stated on the UK Government website [7]. Nodes represent SPI-M contributors and edges represent one or more co-authored publications between contributors listed in PubMed. Colours represent communities of densely connected researchers identified using the spinglass algorithm [9,10]. London School of Hygiene and Tropical Medicine: yellow; Imperial: green; Warwick/Manchester/Lancaster/Bristol/Exeter: orange; Oxford: light blue; PHE/Cambridge: dark blue. Contributors listed online with no connections are not shown (16 individuals).

The functionality and productivity of SPI-M-O has depended to some extent on pre-existing relationships within the field of infectious disease modelling. Many of SPI-M-O contributors have collaborated over many years, and although there are broad groupings of modellers, there are many between-group collaborations (figure 1). The common theoretical underpinning and shared language allows for immediate assessment of work with discussions that assume a firm understanding of disease modelling and focus on technical details.

## 3. What is infectious disease modelling and why is it useful?

‘Disease predictions have reached epidemic proportions’ *Predicting the unpredictable*, Medley [11, p. 1663].

**Figure 2.**
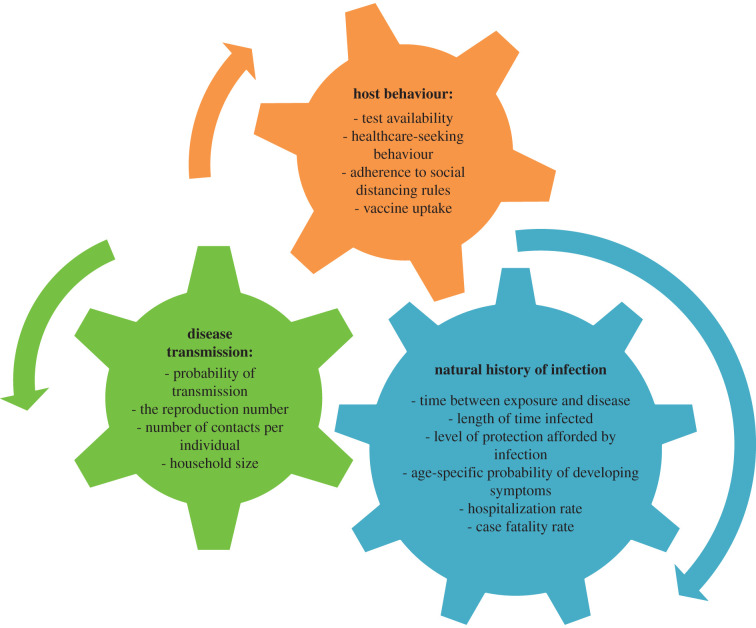
Key components of disease transmission models, and the contributing inputs and parameters. (Online version in colour.)

Infectious disease modelling is the mathematical description of how an infectious disease will spread in a population [12,13]. Unlike statistical modelling, disease modelling involves building a mechanistic description of the epidemic processes, incorporating knowledge of pathogen biology, disease natural history in a host, routes of transmission between hosts and host behaviour (figure 2). The power of disease modelling lies in combining these known factors to assess epidemic drivers and produce predictions. Its limitations can result from relying on essential quantities that have yet to be measured or are difficult to measure.

### (a) Data for models

Infectious disease models typically rely on multiple data sources that are used to constrain model components, these include, but are not limited to: surveillance data (e.g. hospitalizations, confirmed cases [14]) used to monitor epidemic trends and, when informing likely infection events, to infer the timing of transmission between cases (e.g. known infector-infectee pairs, geographical spread with travel history to outbreak locations [15]); demographic data, used to define the population at risk; census and household data, used to characterize household transmission (see [16,17]); and social contact data, essential for predicting the impact of social distancing measures [18].

Early epidemiological data relating to COVID was mainly related to the initial outbreak in Wuhan, collected from China. These early case reports and contact tracing data were used to estimate natural history parameters (such as the time between successive cases estimated in this issue by Challen *et al.* [19]). Very early estimates of the reproduction number, essential to assess the pandemic potential of the new virus, were uncertain, but were greater than 1 and worryingly high—Read *et al*. [15] in this issue produced one of the early estimates. As soon as cases started accumulating in the UK, ‘line lists’ (where each case is captured in a single row of a spreadsheet) could be used for estimating disease transmission parameters such as the reproduction number [19,20] and disease severity, and rapid data sharing agreements had to be established between Public Health England (PHE) and universities. Host behaviour is often difficult to quantify, and it depends on a variety of factors, yet is critical to accurately model an evolving epidemic. SPI-M-O contributors were involved in making sense of the various data streams as well as establishing additional data streams to fill knowledge gaps [21–23].

### (b) Exponential growth is counterintuitive

At its core, disease transmission involves two individuals—an infectious person and a susceptible person who can become infected and infectious themselves. This propagation of infection from one individual to another leads to an exponential growth in cases in the early stages of an epidemic. Although exponentials are common in mathematics and their properties well understood, their implications for control can sometimes be counterintuitive because additions and multiplications are more natural operations when dealing with real data.

**Figure 3.**
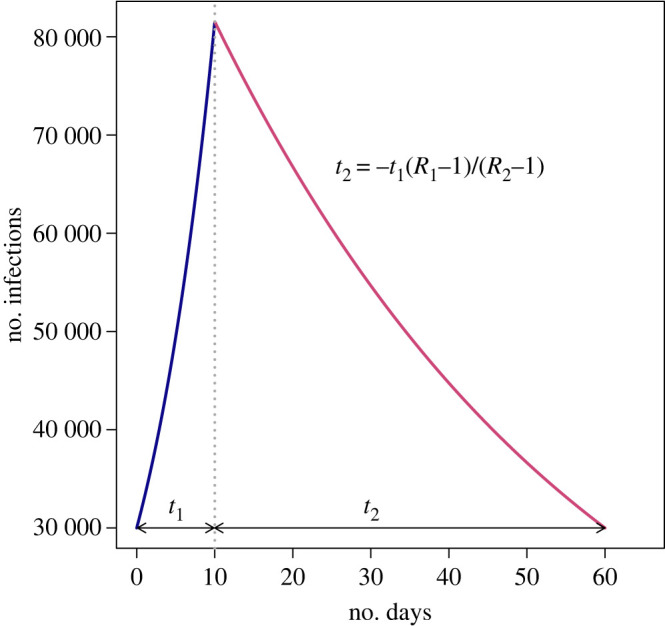
An illustration of the impact of exponential growth when social distancing rules are relaxed and reimposed. (Online version in colour.)

Exponential growth means that the number of cases can quickly get out-of-hand, resulting in increased pressure on hospitals, and require stringent epidemic controls. For example, there was a discussion about relaxing social distancing restrictions over Christmas 2020. At that time, only a relatively small proportion of people were immune to infection, so epidemic growth was still approximately exponential. For $\;t_{1}$ days with no social distancing, the prevalence of infection would increase from $I_{1}$ cases at the start of the relaxation period, to $I_{2}=I_{1}\exp(\gamma (R_{1}-1)t_{1})$ cases at the end of the relaxation period. Plausible values for SARS-CoV-2 are $R_{1}=2$. In this scenario, how many days of lockdown would be required to bring prevalence back to $I_{1}$ cases? Under lockdown, the reproduction number was consistently around 0.8. During lockdown, the number of cases will decline (exponentially) as $I_{2}\exp(\gamma (R_{2}-1)t_{2})$ with $R_{2}=0.8$. The ratio of these two exponents gives the number of lockdown days required for each day of relaxation, $t_{2}=-t_{1\;}(R_{1}-1)/(R_{2}-1)$, which for these plausible values leads to the counterintuitive conclusion that 5 days of lockdown are required for every single day of relaxation (figure 3).

While exponential growth can be disastrous at high prevalence, it is not necessarily worrying in the short term when prevalence is low. For example, at the start of the second UK wave (August–September 2020), reproduction number estimates in the range 1.2–1.5 (or even higher [8]) were sustained for multiple weeks and cases were only slowly creeping up. The reproduction number was generally smaller than that (1.1–1.4 [8]) throughout December 2020, when hospitalizations were rapidly becoming unmanageable.

During exponential growth, the doubling time (time it takes for the number of cases to double) is constant. In this issue, Pellis *et al*. [14] demonstrate that exponential growth with a doubling time of 3 days was observed in the UK and various European Union countries in March 2020. With such a short doubling time, even if countries could double hospital beds overnight, this would only buy them 3 days extra.

## 4. Modelling and policy

‘An 80% right paper before a policy decision is made is worth ten 95% right papers afterwards, provided the methodological limitations imposed by doing it fast are made clear’, What makes an academic paper useful for health policy? Whitty [24, p. 3].

The predictive nature of infectious disease modelling lends itself for use in policy, preparedness and capacity planning and for evaluating policies which might mitigate epidemic spread. The impact of some interventions can be predicted with relatively simple reasoning, but often multiple interacting factors combine to create complex scenarios. In these cases, infectious disease models can be an aid to formalize thinking and quantify qualitatively obvious results. Modelling can be thought of as a mechanism for collating facts and educated guesses into a single framework that can guide policy decisions [25].

Much of the current theory of infectious disease dynamics, taken for granted today, was developed during the early years of the AIDS epidemic—indeed the SPI-M-O chairs Graham Medley and Angela McLean modelled HIV/AIDS transmission in the 1980s, including influential work predicting the number of undiagnosed HIV cases in the UK [26,27]. Since then, modelling has provided evidence used for controlling infectious disease risks in the UK of both humans and animals, including vCJD in the 1990s [28], foot-and-mouth disease outbreaks of cloven hoof animals in 2001 and 2007 [29] and the H1N1 influenza pandemic in 2009 [30], to name a few.

The interaction between modelling and policy is a two-way flow of information. Policy questions shape modelling work, and in return modelling evidence shapes policy. Elizabeth Richards, Tom Irving, Paul Allen, Jen Huynh, Alastair Ikin and other members of the civil service that form the SPI-M-O secretariat are critical to this process. The SPI-M-O secretariat are scientists who work with the SPI-M-O chairs to turn a policy ‘ask’ into a modellable question, and then translate the model results back into relevant evidence and advice. Without this link to decision-makers, SPI-M-O would be an academic forum.

Modellers are encouraged to develop their own independent approaches to avoid groupthink and at least two (but often more) independent analyses are provided for each policy question to aid discussion, explore sensitivity to structural model assumptions and identify inaccuracies, thereby increasing the robustness of SPI-M-O consensus statements. The secretariat and chairs made an active decision not to combine model outputs quantitatively, apart from the medium-term projections and the reproduction number, but rather to use the modelling combined with understanding to generate the policy-relevant consensus. Outcomes are compared to modelling *post hoc*, as an extra validation step.

The speed of the COVID-19 pandemic and the resulting rapidly changing policy landscape calls for modelling evidence to be generated under extreme time pressure. In normal times, it is common for complex models to be developed over six months or even several years. However, during the COVID-19 emergency, models were set up and started generating results in days. The majority of models were not developed from scratch, but relied on existing frameworks—for instance, Danon *et al*. [17], who re-purposed a spatial model of influenza transmission. Complex modelling can reveal truths that are obvious once they have been demonstrated—like the fact that even with vaccination, the number of people who are still susceptible to infection could result in substantial ongoing transmission. Alongside detailed models, basic insights and simple modelling approaches can influence policy by providing a qualitative understanding into transmission dynamics, for example, final size calculations presented by Gog & Hollingsworth to SAGE in February 2020 [31].

## 5. Publishing during a pandemic

The constantly evolving situation and rapid turnaround of modelling evidence are incompatible with the majority of current publishing mechanisms. Policy advice is often needed within days. By contrast, in normal times, scientific manuscripts are peer reviewed over a period of months (although there are beginning to be alternative models involving open peer review, in journals such as F1000 and Wellcome Open Research). Peer reviewing is time consuming and almost completely without credit. During an epidemic emergency, scientific results must be shared immediately and widely, and during 2020, pre-print manuscripts, not yet peer reviewed, became the *modus operandi* for communicating the latest findings. Although pre-prints allowed results to be published rapidly, they lack the quality assurance that peer review, albeit imperfectly, offers. In this regard, the Royal Society's Rapid Assistance in Modelling the Pandemic (RAMP) initiative took on the massive, essential task of rapidly reviewing pre-print manuscripts.

A further conflict between policy and academic impact arises because research conducted in response to policy questions may not be substantial enough to be published as a standalone manuscript. Early in the first wave, SAGE papers and SPI-M-O consensus statements started being published online on the government website [5].

In addition to public health needs, the pace of traditional publishing meant that modelling papers were most likely out-of-date by the time they had gone through peer review. New evidence had come to light, new data had been generated, new papers had come out and it is not practical for scientists to continually update their results in an evolving situation. Therefore, unlike publication in normal times, the time lost during a single rejection from a journal could render the paper out-of-date and unlikely to be published elsewhere. This constantly shifting landscape leads to a tension between generating academic output and providing evidence for public health and government [32], especially with increasing demands for transparency in the scientific evidence behind policy decisions.

Traditional academic output is also at odds with the importance of reproducible and independently reproduced findings. A single model that predicts a large number of cases is of limited use for policy making. Multiple independent predictions are required for robust conclusions to be made. Yet, scientific credit is usually given to the first group to publish a result, not necessarily the scientists who verify findings. Parallel results are hugely important for decision-making, yet policy relevance does not guarantee publication.

## 6. Ensuring transparency and reproducibility

While infectious disease epidemiology has a long-standing tradition of using mathematical modelling and statistical analysis as tools for understanding and predicting disease dynamics, the production of free, open-source tools implementing these approaches is but a recent trend, which has lacked support and recognition until now [32,33]. As a result, the culture of code-sharing in infectious disease modelling is still in its infancy.

In an attempt to enforce scientific reproducibility, peer-reviewed journals are now increasingly requiring code implementing new models to be shared publicly. However, the issue of code-sharing goes beyond publication when said code is used for informing public health policies, as pointed out in the recent debate sparked by the code release of a simulation model used by SPI-M [34]. To inform decision-making as best as possible, scientific evidence needs not only to be based on reliable data and sound models: it requires these two elements to be assembled correctly in bug-free software implementations.

The Office for National Statistics provides an excellent framework for data analysis supporting decision-making, outlining requirements for data analyses to be reproducible, auditable and assured [35]. The work presented at SPI-M was typically validated through two processes. First, rapid peer review of the methodology and data presented in sometimes detailed reports was made by other SPI-M members. Second, most SPI-M results were obtained by effectively combining results from different research groups, using a variety of approaches and often overlapping data sources. Less emphasis was put on ensuring scientific reproducibility, although some groups certainly implemented routine code checks and reviews internally, and usually shared their code on public repositories such as GitHub.

For this issue, we promoted transparency by encouraging all authors to share publicly documented code and data whenever possible. We appreciate all of our contributors' efforts towards improving the reproducibility of the modelling work informing the response to COVID-19, and acknowledge this is but a step towards perfect auditability.

## 7. Putting this special issue together

The motivation for producing this Special Issue included transparency, posterity and providing a mechanism to publish work that shaped policy. Our criteria for inclusion in the Special Issue was that work had been presented at SPI-M-O or SAGE and/or had been used as evidence during policy making. All SPI-M-O contributors were invited to submit their work, and the issue contains representations from major modelling groups involved in SPI-M-O, including the Universities of Lancaster, Warwick, Manchester, Bristol, Cambridge, Oxford, Exeter, Edinburgh, the London School of Hygiene and Tropical Medicine (LSHTM) and Public Health England.

**Figure 4.**
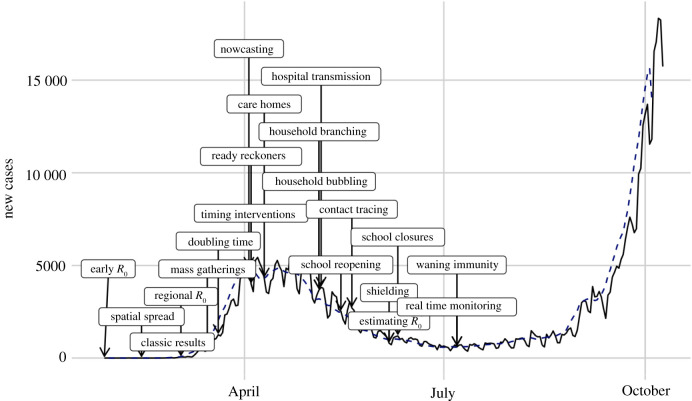
COVID-19 epidemic curve with Special Issue papers marked on, indicating when the work was developed.

The topics covered in this Special Issue provide insight into the detailed evidence that is behind many policy decisions for the UK and its constituent nations. Figure 4 illustrates the approximate timeline of the contributions in the issue, which were mainly during the first wave of COVID-19 epidemic in the UK (January–July 2020).

The issue covers early models that were developed for the UK (figure 4), often with limited data and initially relying on SARS-1-like parameters [15] and theoretical insights [31]. It includes models that are used in the ongoing overview of the epidemic with weekly consensus estimates of the Reproduction number [19,20], short term and medium-term projections [36] and real-time data stream monitoring [37] all playing a part. There are time-sensitive, changing policy questions, such as the impact of mass gatherings [38], reopening schools in May 2020 [39–41], the introduction of support bubbles [42] or the impact of contact tracing and lockdown [43]. We have evidence that drove the understanding of nosocomial and care home transmission [44,45], the importance of segmenting and shielding [46] as well as the possible impact of waning immunity [47]. The breadth of these topics reflects the experience of the modelling community involved in the response in the UK.

We made the decision not to ask authors to update their analyses to include the latest data and latest understanding of the underlying biology and transmission processes, as would normally be requested in traditional journals. This was done in order to provide a record of the work as it was presented and used in real-time, rather than being updated with the benefit of hindsight. Instead, we gave authors the option to either update their manuscripts, with more analyses or an addendum detailing new developments since publication, or to include an additional ‘in-context’ page that describes how the analysis was used to inform policy as well as any important developments that occurred since the piece of work was conducted.

## 8. Summary and legacy

### (a) ‘Tell me why I am wrong’, SPI-M-O unofficial motto, 2020–2021

The COVID-19 pandemic that started in 2020 was extraordinary for many reasons. It was the first time in living memory that social distancing measures were applied on a global scale, and it has probably changed the way we will respond to infectious diseases in the future. The year 2020 was also an extraordinary year for science and infectious disease modelling. COVID-19 propelled infectious disease modelling to the centre of political and general conversation, and communication to non-specialists became an overnight skill required of disease modellers.

Infectious disease models are useful for elucidating epidemic drivers—such as the importance of care homes and hospitals—and predicting the impact of changes in policy, for example, the impact of applying and lifting social distancing measures. Modelling for policy decisions is not the same as regular research in infectious disease modelling. Simple approaches, possibly too simple to be published, are often highly valuable to decision makers. We argue that policy impact should be valued alongside academic impact.

In this introductory article, we attempted to give an overview of the scientific advisory system in the UK, and how modelling contributed to decision-making. It is difficult in absolute terms to quantify the impact of modelling to the national response, and whether there are procedures that could be improved for the next pandemic. The way SPI-M-O operates evolved during 2020—starting with a small number of modellers and expanding to around 50 modellers regularly attending the weekly meetings. This plurality of opinion was key to generating robust and reliable advice. When a consensus view emerged, we could have confidence in it; failure to reach consensus was a reflection of the uncertainty of the situation, and held equivalent value. This plurality needs to be actively supported with funding and training to retain the capacity developed during 2020 and allow for a wide range of views and approaches.

Our ultimate aim in collating this theme issue was to provide a single place where multiple contributions from SPI-M-O could be presented as a collection. We wanted to provide documentation, transparency and acknowledgement of the huge amount of work that was carried out, mainly by scientists who volunteered their time and expertise on top of their regular academic duties. Contributing to SPI-M-O was a privilege and, albeit exhausting, a rewarding and unique experience.

## Data accessibility

This article has no additional data.
